# Flexible, Transparent and Conductive Metal Mesh Films with Ultra-High FoM for Stretchable Heating and Electromagnetic Interference Shielding

**DOI:** 10.1007/s40820-023-01295-z

**Published:** 2024-01-22

**Authors:** Zibo Chen, Shaodian Yang, Junhua Huang, Yifan Gu, Weibo Huang, Shaoyong Liu, Zhiqiang Lin, Zhiping Zeng, Yougen Hu, Zimin Chen, Boru Yang, Xuchun Gui

**Affiliations:** 1 State Key Laboratory of Optoelectronic Materials and Technologies, School of Electronics and Information Technology, Sun Yat-Sen University, Guangzhou, 510275 People’s Republic of China; 2 Guangdong Provincial Key Laboratory of Materials for High Density Electronic Packing, Shenzhen Institutes of Advanced Technology, Chinese Academy of Sciences, Shenzhen, 518055 Guangdong People’s Republic of China; 3 School of Materials Science and Engineering, Sun Yat-Sen University, Guangzhou, 510275 People’s Republic of China

**Keywords:** Metal mesh, Transparent conductive film, Stretchable heater, Electromagnetic interference shielding

## Abstract

Despite the growing demand for transparent conductive films in smart and wearable electronics for electromagnetic interference (EMI) shielding, achieving a flexible EMI shielding film, while maintaining a high transmittance remains a significant challenge. Herein, a flexible, transparent, and conductive copper (Cu) metal mesh film for EMI shielding is fabricated by self-forming crackle template method and electroplating technique. The Cu mesh film shows an ultra-low sheet resistance (0.18 Ω □^−1^), high transmittance (85.8%@550 nm), and ultra-high figure of merit (> 13,000). It also has satisfactory stretchability and mechanical stability, with a resistance increases of only 1.3% after 1,000 bending cycles. As a stretchable heater (*ε* > 30%), the saturation temperature of the film can reach over 110 °C within 60 s at 1.00 V applied voltage. Moreover, the metal mesh film exhibits outstanding average EMI shielding effectiveness of 40.4 dB in the X-band at the thickness of 2.5 μm. As a demonstration, it is used as a transparent window for shielding the wireless communication electromagnetic waves. Therefore, the flexible and transparent conductive Cu mesh film proposed in this work provides a promising candidate for the next-generation EMI shielding applications.

**Supplementary Information:**

The online version contains supplementary material available at 10.1007/s40820-023-01295-z.

## Highlights

- A transparent, conductive, and flexible metal mesh film has been developed by a low-cost, uniform self-forming crackle template and electroplating strategy.
- The Cu mesh films show an ultra-low sheet resistance (0.18 Ω □^−1^), high transmittance (85.8%@550 nm), high figure of merit (> 13,000), excellent stretchability and mechanical stability.
- The metal mesh film can be used as a flexible heater and electromagnetic interference shielding film (40.4 dB at 2.5 μm).

**Supplementary Information:**

The online version contains supplementary material available at 10.1007/s40820-023-01295-z.

## Graphical abstract

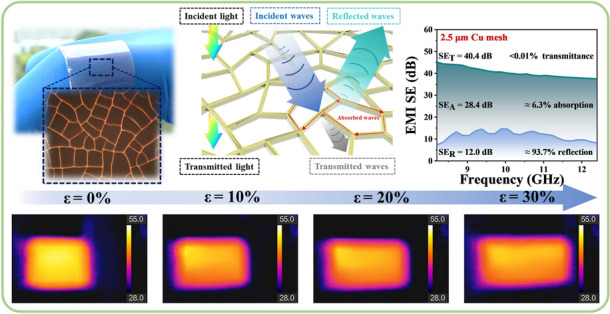

## Introduction

Transparent conductive films (TCFs) play a crucial role in electronic conductive layers and windows in touch-screens display [1–4], solar cell [5, 6], wearable electronic and communication devices [7–9]. Notably, indium tin oxide (ITO) has emerged as a successful material for display touch screens, owing to its excellent figure of merit (FoM), which is a crucial metric to evaluate the comprehensive optoelectronic property of TCFs [10]. However, the traditional ITO-based TCFs suffer from the inherent fragility, which hinders their application in flexible and transparent devices [11, 12]. Consequently, extensive research endeavors have been dedicated to exploring alternative materials and structures to fabricate flexible TCFs, such as carbon nanotubes (CNTs) [13, 14], graphene [15, 16], metal nanowires [17–19], transition metal carbide/carbonitride (MXene) [20–22], and metal mesh [2, 23–26]. Despite the satisfactory advances, some inevitable issues remain for these materials. For example, it is difficult for TCFs based on CNTs or silver nanowires to achieve high FoM and flexibility simultaneously, due to junction resistance and bundling characteristics [13, 17]. Compared to other TCFs materials, the metal mesh can achieve the highest FoM and mechanical stability because of the continuous and junction-free metallic network. Therefore, metal mesh is considered as one of the best candidates for flexible TCFs materials. So far, metal mesh films have primarily been prepared by micro-nanofabrication technique with high-cost and complexity fabrication process, including roll to roll imprinting [23, 24], inkjet printing [27] and ultraviolet (UV) lithography [28, 29]. For instance, the Cu mesh fabricated by roll-to-roll imprinting and electroplating has achieved a FoM of over 10,000, and maintained stable resistance under 50% tensile strain [24]. In addition, the crackle template method can also fabricate flexible metal mesh. Compared to previous methods, crackle template method has lower process and equipment requirements. In addition, it enables the fabrication of metal mesh films on substrates with various shapes [30]. However, compared to the metal mesh prepared by micro-nanofabrication technique, the FoM of the metal mesh prepared by the crack template method is usually lower [2, 30, 31]. For example, the silver mesh film fabricated by crackle template method has a FoM of only 360, which is significantly less than the preceding techniques [2]. Therefore, there is still a challenge to explore low-cost and scalable approaches for achieving flexible, high-performance metal mesh films.

The high conductivity of TCFs provides potential applications for electromagnetic interference (EMI) shielding in wireless electronics and communication equipment [32, 33]. However, the thickness of conductive materials for most of TCFs has a substantial impact on optical transmittance, making it challenging to increase EMI shielding effectiveness (SE) without reducing the optical transmittance. For instance, 2.3 nm thickness MXene film shows an EMI SE of 1.0 dB in the X-band with high optical transmittance (92%@550 nm). Although the EMI SE reaches 10.7 dB with the thickness increasing to 20.7 nm, the transmittance drops to 42% [34]. However, compared to other TCFs, the trade-off between optical transmittance and EMI SE has a less effect on metal mesh. Since the line pitch of metal mesh is between the wavelengths of visible light and microwaves, it means that visible light can pass through the opening area while the microwave is blocked. Thus, through enlarging the opening area and increasing the thickness, the metal mesh film can achieve simultaneous increase in transmittance and EMI SE. For instance, the silver mesh film fabricated by lithography exhibits an EMI SE of 58.4 dB in the X-band with the transmittance of 83% [28]. Although the silver mesh film maintains the high transmittance and achieves over 99% EM waves shielding, the stretchability and mechanical stability are not obtained due to it is fabricated on a glass substrate. Therefore, great effort is still needed to achieve a balance of flexibility, high EMI SE and transmittance for potential practical transparent EMI shielding window in wireless electronics.

Herein, we demonstrate a flexible, transparent and conductive Cu mesh film based on uniform self-forming crackle template and electroplating strategy. It is worth noting that the Cu mesh film based on this method shows an ultra-low sheet resistance (0.18 Ω □^−1^), high transmittance (85.8%@550 nm), and ultra-high FoM (> 13,000). As a stretchable heater, it can generate temperature over 110 °C at 1.00 V supplied voltage within 60 s, and only 4 °C drops at 0.50 V under 30% tensile strain. Meanwhile, the Cu mesh film (2.5 μm) demonstrates a high EMI SE of 40.4 dB within X-Band region (8.2–12.4 GHz). What’s more, the EMI SE remained basically stable (only decreased 1.6 dB) after 1,000 bending cycles. As a demonstration for transparent EMI shielding application, the Cu mesh film realizes the shielding of wireless communication electromagnetic waves. The flexible and transparent conductive film proposed in this work provides a promising candidate for cost-effectiveness, high-EMI SE and transparent electromagnetic shielding windows.

## Experimental Section

### Fabrication of the Cu Mesh Film

Firstly, crackle lacquer (Tianmai, China) was dispersed in deionized water and magnetic stirred for 1 h to obtain uniformly dispersed water-based acrylic emulsion at different concentrations (85%–100%). Afterwards, the emulsion was spin-coated at different speeds (4,000–5,000 rpm) for 15 s on the Ni sheet (70 nm) prepared by E-beam evaporator. After the templates with different thicknesses (1–4 μm) were obtained, which were placed into various sizes culture-dishes (10 × 10 or 15 × 15 cm^2^) and dried at 35 °C for 5 min. The Cu plating solution was prepared (70 g L^−1^ CuSO_4_·H_2_SO_4_, 240 g L^−1^ H_2_SO_4_, 5 mg L^−1^ SPS, 50 mg L^−1^ PEG) for electroplating. Next, Cu^2+^ was deposited in the microgrooves on the Ni sheet with the current density of 20 mA cm^−2^ for 30–90 s. The specific preparation parameters for each Cu mesh of different coverage ratios and thicknesses are shown in Tables S1 and S2, respectively. The sacrificial template was removed by the mixture of acetone and KOH. After covering the Cu mesh with a certain quality of PDMS (30 mg cm^−2^) and cured at 50 °C for 2 h, a Cu mesh film semi-embedded into PDMS was obtained.

### Characterization

The metal mesh surface morphology was characterized by optical microscopy (Carl Zeiss, Axio CSM 700) and scanning electron microscopy (Hitachi, S-4800). The sheet resistances were measured by 4-point probes resistivity measurement system (4 probes tech, China), and transmittance in visible spectrum was measured by UV–Vis spectrophotometer (Hitachi, U-4100). The statistical coverage ratio and average line width were calculated by software (Image-J), and the thicknesses of the samples were measured by step profiler (KLA instrument, Alpha-step D-300). Next, the mechanical endurance was tested on a universal testing machine (Instron, 5943), and the corresponding resistance changes were measured by source measure unit (Keithley 2400), which can also apply direct current power for Joule heating. The temperatures were determined, and the infrared images were captured by infrared thermal imager (Hikmicro, K-20). The EMI SE was determined by using a vector network analyzer (N5232, VNA, Keysight) based on the waveguide method within 8.2–12.4 GHz region (X-band).

## Results and Discussion

### Fabrication and Characterization of the Transparent Conductive Cu Mesh Films

**Fig. 1:**
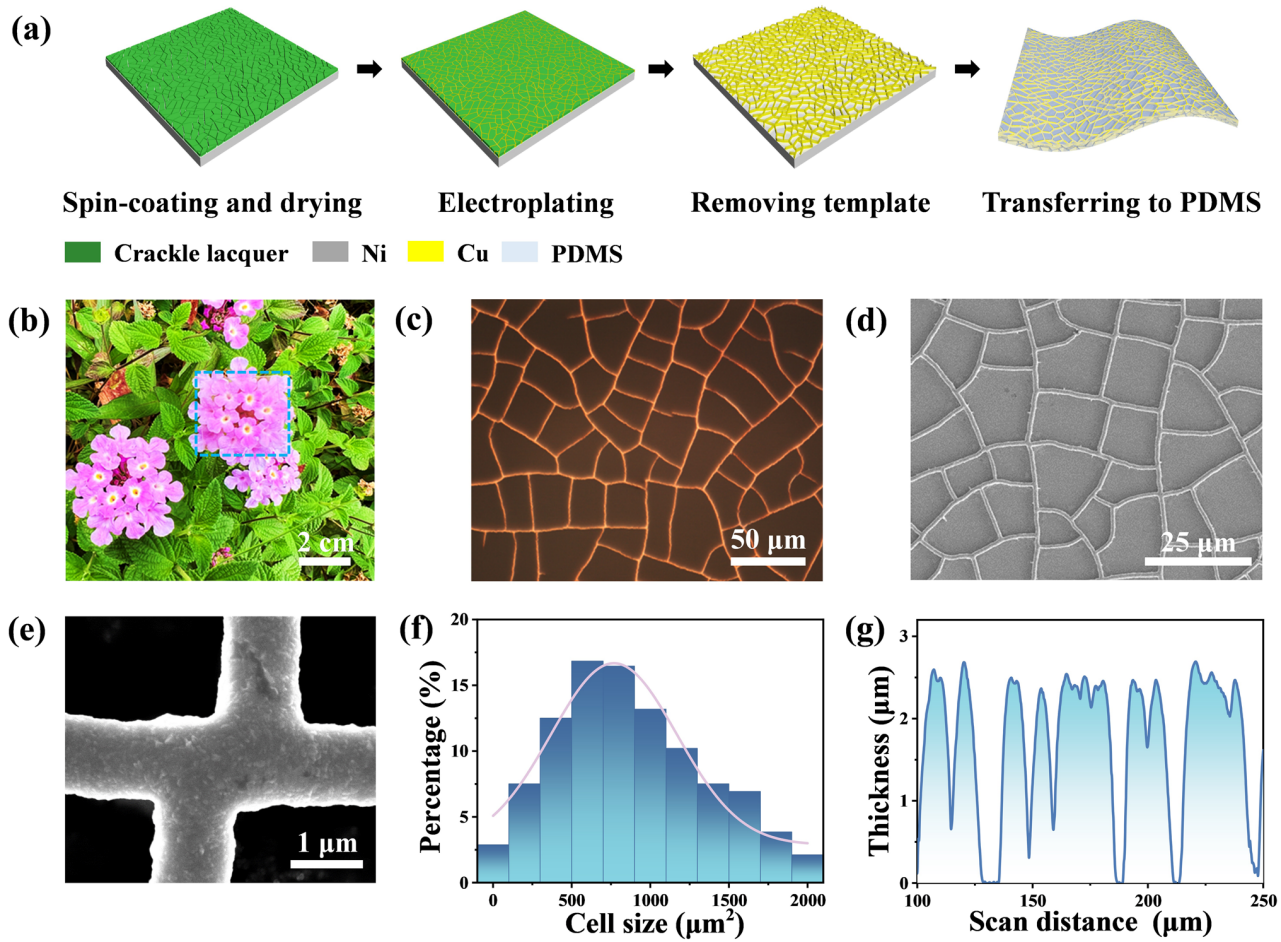
Fabrication and characterization of Cu mesh film. **a** Schematic diagram of the fabrication process for a Cu mesh film. **b** Digital photograph of a 3 × 2.5 cm^2^ Cu mesh film. **c** OM image of Cu mesh film. **d, e** SEM images of the Cu mesh. **f** Cell size distribution of Cu mesh. The number of statistical cells exceeds 1000. **g** Thickness distribution of a Cu mesh measured by step profiler

The transparent, conductive Cu mesh film is fabricated by a self-forming crackle template method and Cu electroplating technique. As demonstrated in Fig. 1a, a specific concentration of crackle lacquer, which is mainly composed of water-based acrylic emulsion, is spin-coated on a nickel (Ni) sheet. The crackle template develops under specific drying circumstances [35, 36], and then Cu^2+^ is deposited in the microgrooves by electroplating to form a Cu mesh (Fig. S1). After removing the sacrificial layer, curing PDMS casted onto the mesh and peeling it off the Ni sheet, the Cu mesh film semi-embedded into PDMS is obtained. Detailed fabricating process and parameters are described in experiment section. The transmittance of the Cu mesh film allows for clear visibility of a flower (Fig. 1b). The optical microscopy (OM) and scanning electron microscopy (SEM) images demonstrate the Cu mesh film remain continuity and integrity after transferring (Fig. 1c, d). The small line width and large cell size of the Cu mesh film contribute to the high transmittance. As shown in Fig. 1e, the intersection and line width of the Cu mesh are about 1.0 μm. The cell size of Cu mesh is mainly varying from 400 to 1,200 μm^2^ (Fig. 1f). In addition, the thickness of the Cu mesh is very uniform, as shown in Fig. 1g.

**Fig. 2:**
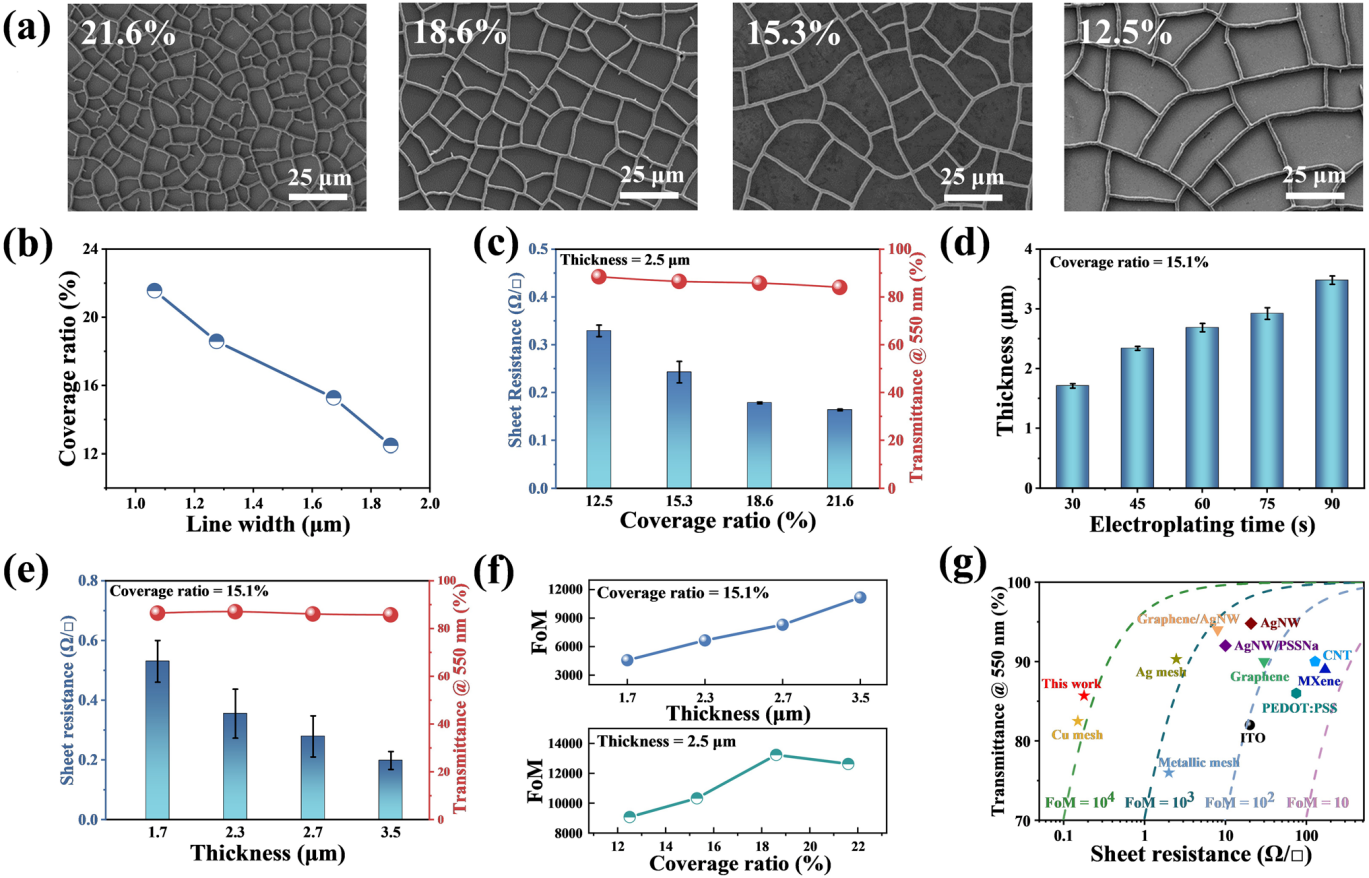
Optoelectronic characterization of Cu mesh films with different coverage ratios and thicknesses. **a** SEM images of Cu mesh with different coverage ratios. **b** Relationship between line width and coverage ratio of the Cu mesh. **c** Sheet resistance and optical transmittance at 550 nm of the films with different coverage ratios. **d** Thicknesses of the Cu mesh at different electroplating time. **e** Sheet resistance and optical transmittance at 550 nm of the films with different thicknesses. **f** FoM of the Cu mesh film with different thicknesses (up), and coverage ratios (down). **g** Comparison of sheet resistances and transmittances between different TCFs, which reported in the literature and this work. The dashed line indicates that FOM is equal to a specific value listed in the figure. Details about the literatures are listed in Table S3

Cu mesh films with different line widths, cell sizes and coverage ratios can be obtained by adjusting and controlling the solvent content of the crackle lacquer and drying conditions of the crackle template. As the solvent content increases, the cell size and line width of mesh decrease due to the greater capillary pressure generated by the higher solvent evaporation rate [36]. Furthermore, the temperature of the drying environment is fine-tuned to avoid the comprehensive performance decline caused by template separating with the substrate due to the high evaporation rate. The SEM images and OM images of the Cu meshes with different line widths and spacings are shown in Figs. 2a, S2 and S3. Through Image-J software analysis, the coverage ratio of Cu mesh (the proportion of the area covered with Cu to the whole area) can be achieved from 12.5% to 21.6%, and the corresponding line width changes from 1.9 to 1.1 μm (Fig. 2b). With the line width increases, the main cell size of different Cu meshes increases from 500 to 4,000 μm^2^, as shown in Fig. S4. In order to verify the effect of coverage ratio and line width on the optoelectronic properties of the film, the thickness of the Cu meshes are controlled at the same level (Figs. S5 and S6) by adjusting the electroplating time. Since the aperture of the Cu mesh is significantly larger than the wavelength of visible light, the transmittance of the Cu mesh is primarily determined by the coverage ratio. As shown in Fig. 2c, the transmittance (@550 nm) of the Cu mesh film decreases from 88.5% to 84.0% with the coverage ratio increase from 12.5% to 21.6%. According to previous literature reports [29], the sheet resistance of Cu mesh on a scale larger than the period of the gird can be expressed by $R_{\square } = \xi \frac{{\rho}}{{t\cdot r_{c} }}$, where *ξ* is a correction factor which depends on deposition conditions and lattice properties, as well as line and junction imperfections, *ρ* is the electrical resistivity of the metal, *t* is the thickness and *r*_*c*_ is the coverage ratio. In this work, the sheet resistance of the Cu mesh decreases from 0.33 to 0.16 Ω □^−1^ with the coverage ratio increase from 12.5% to 21.6% (Fig. 2c).

In addition, the thickness of Cu mesh is also a key factor affecting the optoelectronic performances. The electroplating time is directly proportional to the thickness of the Cu mesh film. The OM images of Cu mesh prepared in the same template at different thicknesses by adjusting the electroplating time are shown in the Fig. S7. Furthermore, the cross section of Cu meshes are shown in Fig. S8. It is worth mentioning that too long electroplating time will cause too much Cu^2+^ deposition to overflow the cracks of the template, resulting in an increase in line width and a decrease in light transmittance [37]. The thickness of the Cu mesh increases from 1.7 to 3.5 μm when the electroplating time increases from 30 to 90 s (Figs. 2d and S9). As shown in Figs. 2e and S10, the sheet resistance decreases from 0.53 to 0.19 Ω □^−1^ and the transmittance (@ 550 nm) changes from 86.4 to 85.7% with the thickness increasing from 1.7 to 3.5 μm, respectively.

Typically, conductivity and optical transmittance is trade-off for TCFs [2, 38]. Hence, FoM is widely used to compare the comprehensive optoelectronic properties of TCFs. It is defined as the ratio of electrical conductance (*σ*_*dc*_) to optical conductance (*σ*_opt_) and calculated by using the commonly accepted equation below [23, 37]:

1 $${\text{FoM}} = \frac{{\sigma_{dc} }}{{\sigma_{{{\text{opt}}}} }} = \frac{{Z_{0} }}{{2R_{S} \left( {\frac{1}{\sqrt T } - 1} \right)}}$$

where *Z*_*0*_ is the impedance of free space (*Z*_0_ = 377 Ω), *R*_*s*_ is the sheet resistance of TCFs, and *T* is the transmittance at a wavelength of 550 nm. The FoM *versus* the thickness (top) and coverage ratio (down) of Cu mesh film are shown in Fig. 2f. The FOM shows an almost linear upward trend, ranging from 4,568 to 11,167. The sheet resistance decreases with the thickness of Cu mesh increasing, while the light transmittance changes little, resulting in the increase of FoM. Interestingly, the effect of coverage ratio on FoM is not an almost linear upward trend, unlike the effect of thickness. When the coverage ratio comes to 18.6% at the thickness of 2.5 μm, the Cu mesh achieve the highest FoM (about 13,232) with the sheet resistance of 0.18 Ω □^−1^ and transmittance at 550 nm of 85.8%. A comparison about optoelectronic properties among other TCFs in recently reported literatures (Fig. 2g, Table S3). The results indicate that the TCFs reported in this work exhibits outstanding comprehensive performance. The FoM of Cu mesh is about two orders of magnitude higher than the other materials, such as ITO [39], silver nanowires [40, 41], graphene [1, 42], CNT [43], MXene [22], and conductive polymer [44]. Meanwhile, the comprehensive performance of the Cu mesh based on uniform self-forming crackle template can exceed the other methods of fabricating metal mesh at the same thickness [2, 24, 28]. Thus, the high optoelectronic performance of the Cu mesh based on the self-forming crackle template with remarkable controllability and scalability is promising for flexible transparent window of wearable electronics.

### Mechanical and Electrothermal Properties of the Flexible Cu Mesh Films

**Fig. 3:**
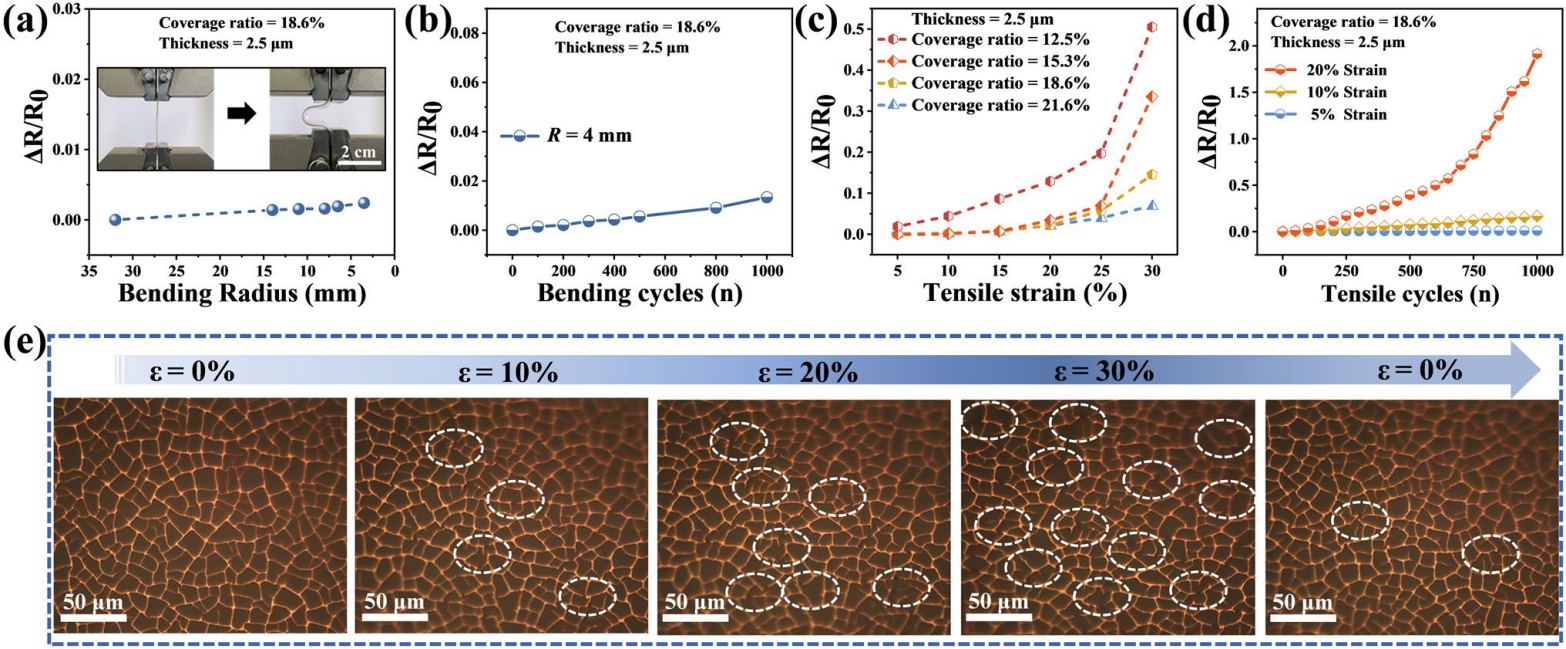
Mechanical properties of the flexible Cu mesh films. **a** Δ*R*/*R*_0_ (*ΔR* is the change in resistance value, *R*_0_ is the initial resistance) of Cu mesh films at different bending radius. Insets are the digital photographs of initial and bending states. **b** Δ*R*/*R*_0_ of Cu mesh film during 1000 bending cycles at the bending radius of 4 mm. **c** Δ*R*/*R*_0_ of Cu mesh films with different coverage ratios at varying tensile strain (*ε*). **d** Δ*R*/*R*_0_ of Cu mesh films during 1,000 tensile cycles at different tensile strains. **e** OM images of a Cu mesh film under different stretching states

The Cu mesh possesses remarkable flexibility and mechanical endurance due to its open-mesh structure, which allows the strips to rotate and accommodate stretching or bending deformations [45]. In addition, the Cu mesh is semi-embedded into the PDMS. Due to the high elasticity of PDMS substrate, the flexibility and mechanical endurance of Cu mesh is further improved [46]. In order to exhibit the flexibility of the Cu mesh film, the relative resistance variation (△*R*/*R*_0_) of which under different bending radius are shown in Fig. 3a, where *R*_0_ is the initial resistance of the film, and △*R* is the change in resistance. The △*R*/*R*_0_ of Cu mesh only increases by 0.25% under a bending radius of 3.5 mm, verifying that the open-mesh structure is beneficial to the flexibility of the Cu mesh. To further show the mechanical endurance of Cu mesh film, the △*R*/*R*_0_ is tested after multiple bending cycles with a radius of 4.0 mm (Fig. 3b). After 1,000 bending cycles, a slight decline of 1.3% in resistance can be observed, indicating the excellent bending endurance of the flexible Cu mesh film. The △*R*/*R*_0_ of Cu mesh films with different coverage ratios as the tensile strain increases from 5% to 30% are shown in Fig. 3c. The larger coverage ratio is provided with better stability because of more intersections. The △*R*/*R*_0_ during 1,000 stretching cycles at different tensile strain are shown in Fig. 3d. The resistance of Cu mesh only changes by 1% at tensile strain of 5%, because the stress is released through grid deformation. However, the resistance changes by more than 16% at 10% strain, indicating difficulties in the recovery of Cu mesh fracture vertex to the initial state under this degree of strain. With the increase in the strain, the Cu mesh failure becomes more serious. When the tensile strain reaches to 20%, the resistance changes close to 200%. As a result, the flexible Cu mesh exhibits high mechanical endurance under small degree strain. In order to further explore the mechanical stability of the Cu mesh, OM images of Cu mesh films fracture under different strain and recovery after 30% strain are shown in Fig. 3e. At 10% strain, most of the meshes expand in the stretching direction, but some intersections are destroyed. As the strain increases to 30%, more break points appear on the Cu mesh, resulting in degradation of electrical performance. However, some destroyed intersections of Cu mesh are unable to reconnect after returning to the initial state, leading to the electrical properties failing to return to their initial state.

**Fig. 4:**
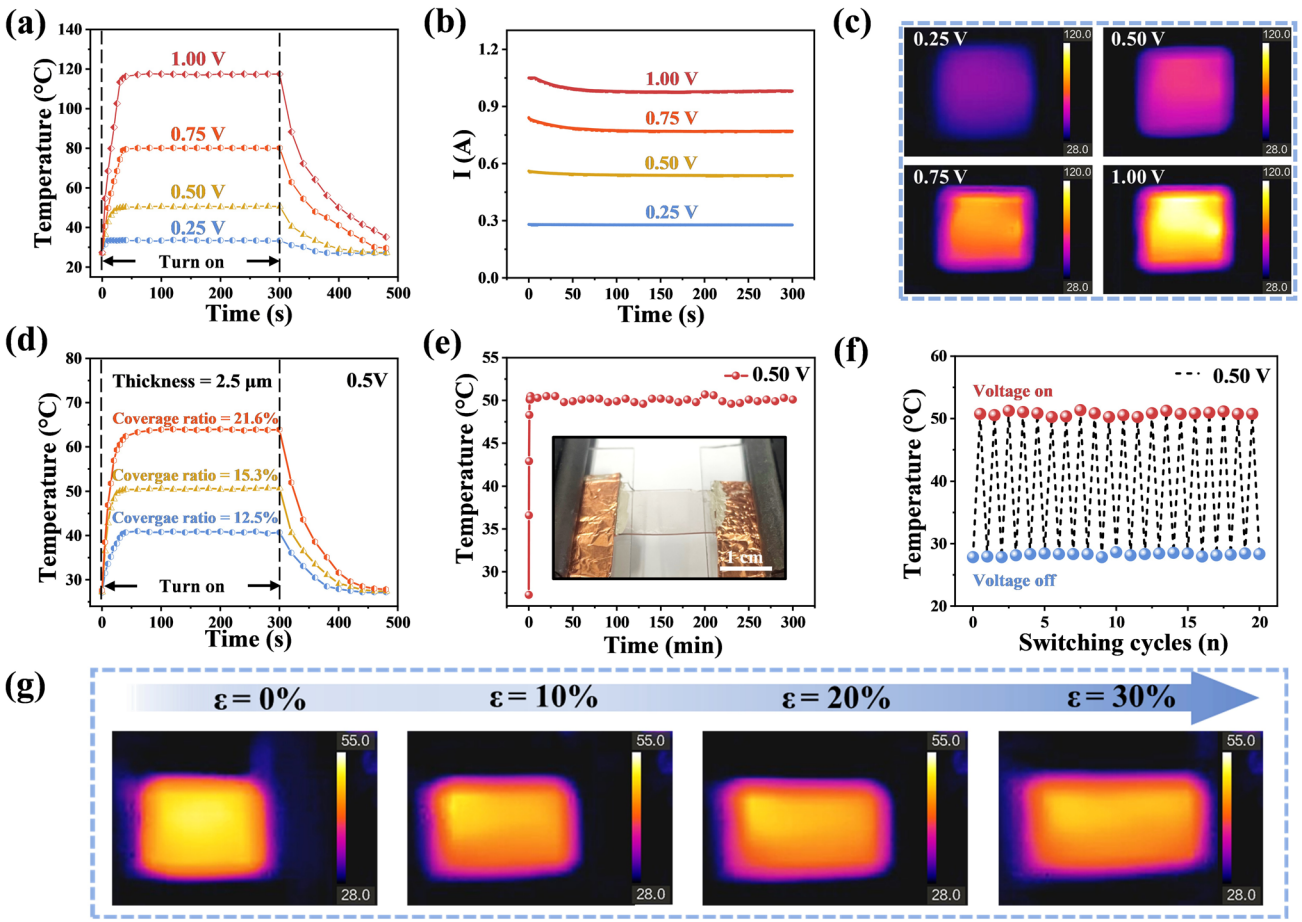
Electrothermal properties of the flexible Cu mesh film (Coverage ratio = 15.3%, thickness = 2.5 μm). **a** Time-dependent surface temperatures of Cu mesh film under different supplied voltages. **b** Corresponding current values to different supplied voltages (Compliance currents = 1.05 A). **c** Infrared images of the Cu mesh film at different supplied voltages. **d** Time-varying surface temperature of the Cu mesh films with different coverage ratios at 0.50 V supplied voltage. **e** Temperature stability of the film during 5 h under continuous 0.50 V voltage supplied. Insets shows that a Cu mesh film is used as a heater. **f** Thermal response of the film under cycle-to-cycle voltage on–off at 0.50 V. **g** Infrared images of the Cu mesh film under different tensile strains (*ε* = 0%, 10%, 20% and 30%)

The excellent optoelectronic conductivity of the Cu mesh film also opens up possibilities in the application of transparent electrothermal devices, such as thermotherapy [47] and defroster heaters [23]. According to Joule’s law, $J = \frac{{V^{2} }}{R} \cdot t$, where $J$ is the heat generated by the Joule effect, $V$ is the applied voltage, $R$ is the resistance of the heaters (Cu mesh film) and $t$ is the time. Thus, the heat generated is directly correlated with the applied voltage and resistance. As shown in Fig. 4a, the highest saturation temperature of the films (coverage ratio = 15.3%, thickness = 2.5 μm, R_S_ = 0.26 Ω □^−1^) increase from 33 to 50, 80 and 110 °C as the applied voltages increase from 0.25 V to 0.50, 0.75 and 1.00 V, respectively. The temperature data was taken while the heating film was free-standing in the air, and the covering area of the heating film is 1.5 × 1.5 cm^2^. The Cu meshes possess a relatively fast heating up time, and the Cu mesh films rise from room temperature to the saturation temperature within 60 s at different applied voltages. The corresponding current value can maintain at the saturation temperature after decreasing to the constant value (Fig. 4b), following the positive temperature coefficient (PTC) of Cu. The infrared radiation images for four different applied voltages are shown in Fig. 4c, which indicate the Cu mesh film shows a satisfactory thermal uniformity especially in a high applied voltage.

According to Joule’s law, a lower resistance allows for greater heat conversion at a given applied voltage. The electrothermal properties of Cu mesh films with different coverage ratios (sheet resistance) are investigated in Fig. 4d. The saturation temperature of Cu mesh film (2.5 μm) can reach 64, 50 and 40 °C at 0.50 V applied voltage, corresponding to the coverage ratio of 21.6%, 15.3% and 12.5%, respectively. Therefore, the low sheet resistance with large coverage ratio of Cu mesh film is able to improve the saturation temperature of heating film. The heater durability is assessed by applying continuous voltage (0.50 V) to the Cu mesh film with the coverage ratio of 15.3% and thickness of 2.5 μm (Fig. 4e). The saturation temperature of Cu mesh film can remain at about 50 °C for 5 h, demonstrating the excellent durability for long-term stable heaters. Meanwhile, the heater exhibits cycle-to-cycle saturation temperature stability between voltage on and off at 0.50 V applied voltage, as shown in Fig. 4f.

Tensile stability is an essential condition for the application of heaters in the field of human hyperthermia therapy. Hence, it is meaningful to investigate the heating performance of Cu mesh film under tensile strain. The infrared radiation images corresponding to different tensile strains of Cu mesh film are shown in Fig. 4g. The saturation temperature of Cu mesh decreases slowly from 52 to 48 °C with the strain increased to 30% at 0.50 V applied voltage. The resistance and increasing heating area of the Cu mesh changed during the stretching process lead to a decrease in the saturation temperature [24].

### EMI Shielding Performance of the Cu Mesh Film

Highly conductive Cu mesh is also suitable for protecting the electronic components from harmful electromagnetic waves. The performance of a shield against incident EM waves is referred to EMI SE. The value of EMI SE is calculated as the ratio of transmitted power (*P*_T_) to incident power (*P*_I_) on a logarithmic scale, as shown in the equation below [48, 49]

2 $${\text{SE}}_{{\text{T}}} \left( {{\text{d}}B} \right) = - 10 \log \frac{{P_{{\text{T}}} }}{{P_{{\text{I}}} }}$$

**Fig. 5:**
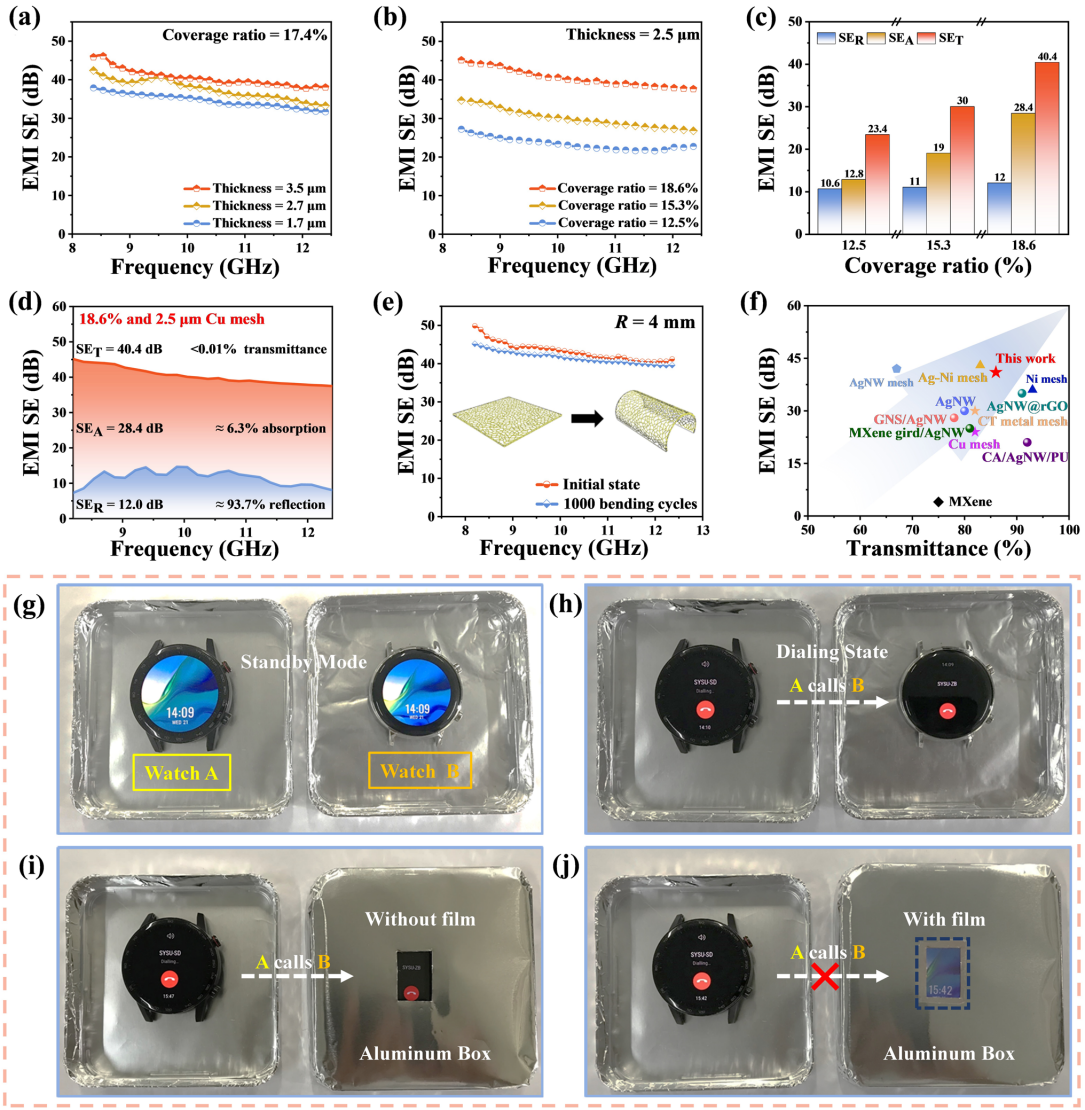
EMI shielding performance of the flexible Cu mesh films. **a** Total EMI SE of the Cu meshes (Coverage ratio = 17.4%) with different thicknesses in the X-band (8.2–12.4 GHz). **b** Total EMI SE of Cu mesh (2.5 μm) with different coverage ratios in the X-band. **c** Average reflection (SE_R_), absorption (SE_A_), and total EMI SE (SE_T_) of the films with different coverage ratios. **d** Contributions of SE_R_ and SE_A_ to SE_T_ for a 2.5 μm and 18.6% coverage ratio Cu mesh film. **e** Total EMI SE of Cu mesh at initial state and after 1000 bending cycles at the radius of 4 mm. Insets are the schematic diagrams of bending. **f** Comparison of the EMI SE and transmittance of this work with other materials in the literature. The details are listed in Table S4. Demonstration for shielding wireless communication electromagnetic waves. **g** Standby mode of two watches. **h** Dialing state of two watches when A was calling to B. **i** State of two watches when A was dialing to B which was placed in an aluminum box with an opening window. **j** State of two watches when A was dialing to B when the Cu mesh film was tightly attached to the opening window

The attenuation of EM intensity occurs through reflection and absorption mechanisms. The electromagnetic shielding mechanism of metal mesh film is mainly reflection, which depends on the interface between two media with different impedances (Fig. S11). The average EMI SE of the Cu mesh film at the thickness of 3.5 μm is 40.7 dB in the X-band, including 13.5 dB of reflection (SE_R_) and 27.2 dB of absorption (SE_A_) (Figs. 5a and S12). More meaningfully, the cell size of the Cu mesh is between the wavelength of visible light and GHz EM waves, it means that visible light can pass through the aperture without attenuation but the GHz EM waves will be shielded. The optical transmittance of Cu mesh is over 85%, which far exceeds other shielding materials in equivalent EMI SE, such as MXene [50–53], cellulose composite [54] and graphene [55, 56]. Furthermore, the EMI SE of the Cu mesh film can be improved by increasing the thickness with a slightly reduction in transmittance. The shielding effectiveness of reflection for various thicknesses film are about 13.5 dB because of the relatively close electrical conductivity (Fig. S12).

The cell size of metal mesh is one of the factors affecting the EMI SE of shielding films [57]. To investigate the impact of the mesh aperture and line width on the EMI SE, the different coverage ratios of Cu mesh films with a thickness of 2.5 μm are tested at the frequency of 8.2–12.4 GHz. The EMI SE of Cu mesh with 18.6% coverage ratio (main cell size of 800 μm^2^) achieved the highest average EMI SE value of 40.4 dB in the X-band. As the main cell size of the film increases from 800 to 4,000 μm^2^, the EMI SE also decreases to 23.4 dB (Fig. 5b). The detailed total SE_T_, SE_R_ and SE_A_ values are shown in Fig. 5c, the SE_R_ of the film shows a slight upward trend with the increase of the coverage ratio. Meanwhile, there is a significant increase in absorption from 12.8 to 28.4 dB. The SE_R_ and SE_A_ of the Cu mesh with 18.6% coverage ratio are 12.0 and 28.4 dB, respectively. The 12.0 dB SE_R_ means that 93.7% of the incident EM wave is reflected on the surface when it penetrates to the Cu mesh film. Also, the 28.4 dB SE_A_ means that 2.3% of the incident EM wave is absorbed (Fig. 5d). Thus, 85.8% of visible light can pass through the open areas of Cu mesh film, but only 0.01% of the incident electromagnetic wave in the X-band can pass through. In general, an increase in coverage ratio and a decrease in cell size can both enhance the EMI SE. The effectiveness of both is verified through simulation using the CST studio suite. As expected, the total EMI SE improves as the coverage ratio increases and the cell size decreases, respectively (Fig. S13). Since the parameters we set are similar to those in the experiment, the variation in cell size plays a significant role in the enhancement of the EMI SE.

In order to demonstrate the satisfactory flexibility and high shielding performance of the Cu mesh film, it is bent at a 4.0 mm radius and stretched at 10% tensile strain for 1,000 cycles respectively. The value of EMI SE drops only 1.6 dB after the bending test, which means that the GHz electromagnetic wave pass through the film only increases by 0.002% (Fig. 5e). In the stretching test, due to the partial fracture of continues mesh structure, it leads to a greater change in the EMI SE than that of bending test. The average value of EMI SE drops by 3.1 dB when it is stretching to 10% tensile strain and 12.7 dB under 30% tensile strain (Fig. S14a). Meanwhile, the average value of EMI SE drops from 35.5 dB to 27.2 dB after cyclic stretching test (Fig. S14b). In a word, Cu mesh films proposed in this work show great potential for application in wearable electronics for shielding harmful electromagnetic waves because of excellent electrical conductivity and mechanical endurance in EMI shielding. The comparison in optical transmittance and average EMI SE within GHz region between the Cu mesh and other flexible transparent conductive films based on graphene, AgNW, MXene and metal mesh are shown in Fig. 5f and Table S4. Compared to other flexible transparent electromagnetic shielding materials [34, 58–63], the Cu mesh exhibits a satisfactory comprehensive performance. In the realm of metal mesh [30, 64–67], the EMI SE of self-forming Cu mesh in this work is superior than that of the lithography technique at the same thickness.

To visually demonstrate the transparency of the Cu mesh film and its shielding effectiveness against electromagnetic waves in the GHz region, two smart watches were utilized for the presentation as shown in Figs. 5g–j. Both watches showed the normal standby state (Fig. 5g), and then watch A was used to call watch B, indicating that the wireless communication electromagnetic waves (2.4 GHz) can be transmitted normally (Fig. 5h). Next, watch B was placed inside an aluminum box with an opening window before being called, which still could pick up communication signals (Fig. 5i). However, after covering the opening window with the Cu mesh film, the watch A was no longer able to communicate with watch B (Fig. 5j), illustrating the Cu mesh film is able to effectively block the transmission of electromagnetic waves. The Cu mesh proposed in this work provides a promising candidate for next-generation transparent and flexible electromagnetic shielding window in wireless communication devices.

## Conclusion

We demonstrate a flexible, high conductive, and transparent Cu metal mesh for stretchable heater and EMI shielding application. The uniform and continuous film is realized by the self-forming crackle template method and electroplating strategy. Through the remarkable controllability and scalability in processing and cost-efficient procedure of this metal mesh film, a Cu mesh film with high optical transmittance of 85.8%, low sheet resistance of 0.18 Ω □^−1^ and high FoM over 13,000 was fabricated. Thanks to the high optoelectronic quality and flexibility, the saturation temperature of Cu mesh can reach over 110 °C at 1.00 V applied voltage within 60 s, and only 4 °C drops at 0.50 V under 30% tensile strain. Remarkably, the Cu mesh film can achieve a satisfactory EMI SE of 40.4 dB and excellent flexibility while maintaining the performance after 1,000 bending cycles. The effective shielding of the wireless communication signal electromagnetic waves indicates that the Cu mesh film has potential applications for practical EMI shielding and next-generation EM-compatible optoelectronic devices.

## Supplementary Information

Below is the link to the electronic supplementary material.

Supplementary file1 (PDF 1879 KB)
