# Supplementary material for: Flexible, Transparent and Conductive Metal Mesh Films with Ultra-High FoM for Stretchable Heating and Electromagnetic Interference Shielding
Source: Nanomicro Lett. 2024 Jan 22;16:92. doi: 10.1007/s40820-023-01295-z (PMC10803711; doi:10.1007/s40820-023-01295-z)
Supplement: Supplementary file 1 — Supplementary file1 (PDF 1879 KB) [file 40820_2023_1295_MOESM1_ESM.pdf]

Supporting Information for

Flexible, Transparent and Conductive Metal Mesh Films with Ultra-High FoM for Stretchable Heating and Electromagnetic Interference Shielding

Zibo Chen[1,#], Shaodian Yang[1,#], Junhua Huang[1], Yifan Gu[1], Weibo Huang[1], Shaoyong Liu[1], Zhiqiang Lin[2], Zhiping Zeng[3], Yougen Hu[2], Zimin Chen[1], Boru Yang[1,*], and Xuchun Gui[1,*]

[1] State Key Laboratory of Optoelectronic Materials and Technologies, School of Electronics and Information Technology, Sun Yat-sen University, Guangzhou 510275, P. R. China

[2] Guangdong Provincial Key Laboratory of Materials for High Density Electronic Packing, Shenzhen Institutes of Advanced Technology, Chinese Academy of Sciences, Shenzhen, Guangdong 518055, P. R. China

[3] School of Materials Science and Engineering, Sun Yat-sen University, Guangzhou 510275, P. R. China

[#] Zibo Chen and Shaodian Yang contributed equally to this work.

*Corresponding authors. E-mail: guixch@mail.sysu.edu.cn (Xuchun Gui); yangboru@mail.sysu.edu.cn (Boru Yang)

Supplementary Figures and Tables

The total EMI shielding effectiveness (SE_T) is the sum of the absorption of electromagnetic energy (SE_A), the reflection (SE_R). Reflectivity (R), absorptivity (A), and transmissivity (T) are determined based on the measured parameters including reflection (S_{11}) and transmission (S_{12}) parameters, the equations are as follows:

$$EMI\ SE_T = SE_A + SE_R \quad (S1)$$

$$R = |S_{11}^2| = |S_{22}^2| \quad (S2)$$

$$T = |S_{12}^2| = |S_{21}^2| \quad (S3)$$

$$A = 1 - R - T \quad (S4)$$

$$SE_T = -10\ logT = -20\ logS_{12} \quad (S5)$$

$$SE_R = -10\log(1 - R) \quad (S6)$$

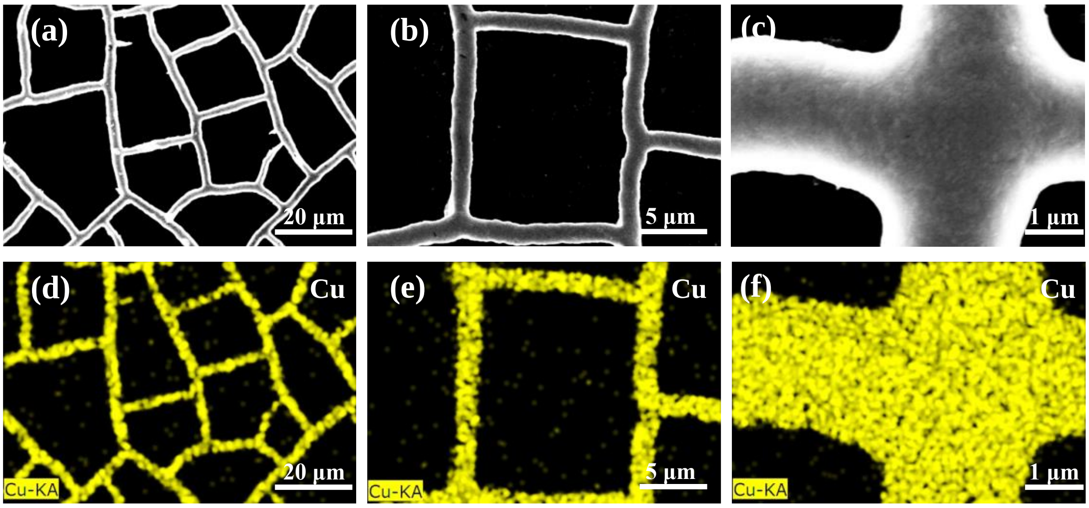

Fig. S1 a-c SEM images of Cu mesh. **d-f** Corresponding EDS mapping results

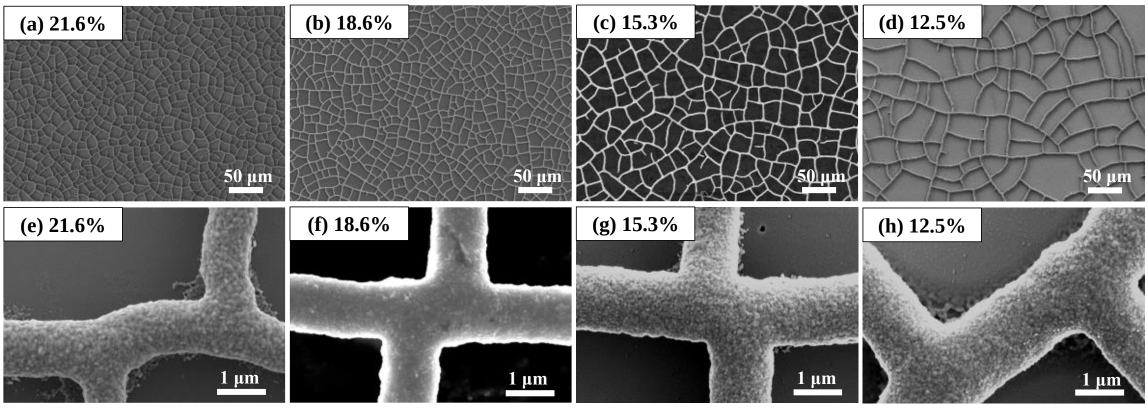

Fig. S2 SEM images of the Cu mesh films with different coverage ratios

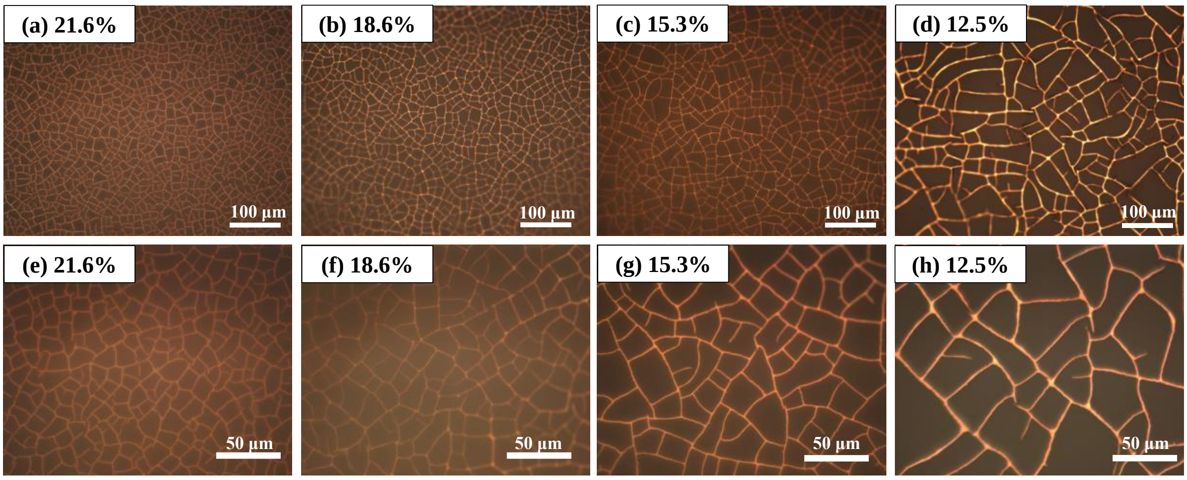

Fig. S3 OM images of the Cu mesh films with different coverage ratios

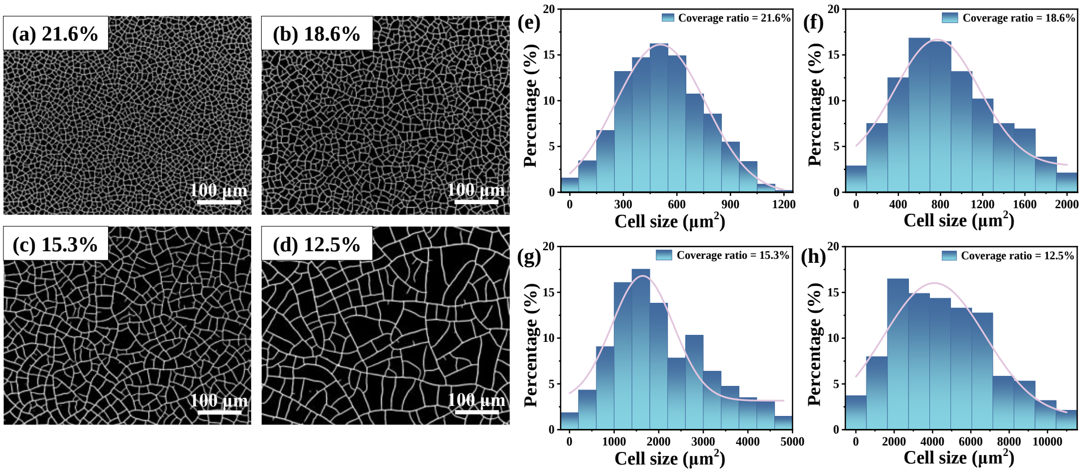

Fig. S4 a-d Images of the Cu meshes processed by image-J (The white areas represent Cu). **e-h** Cell size distributions of the Cu mesh with different coverage ratios

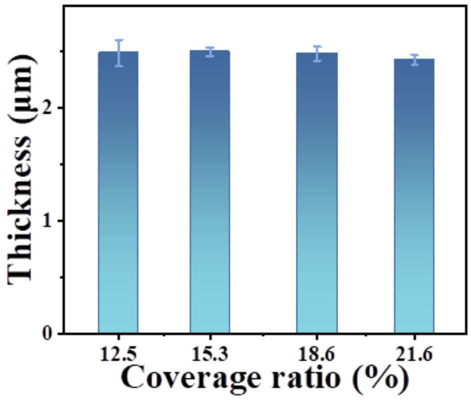

Fig. S5 Thicknesses of the Cu mesh films with different coverage ratios

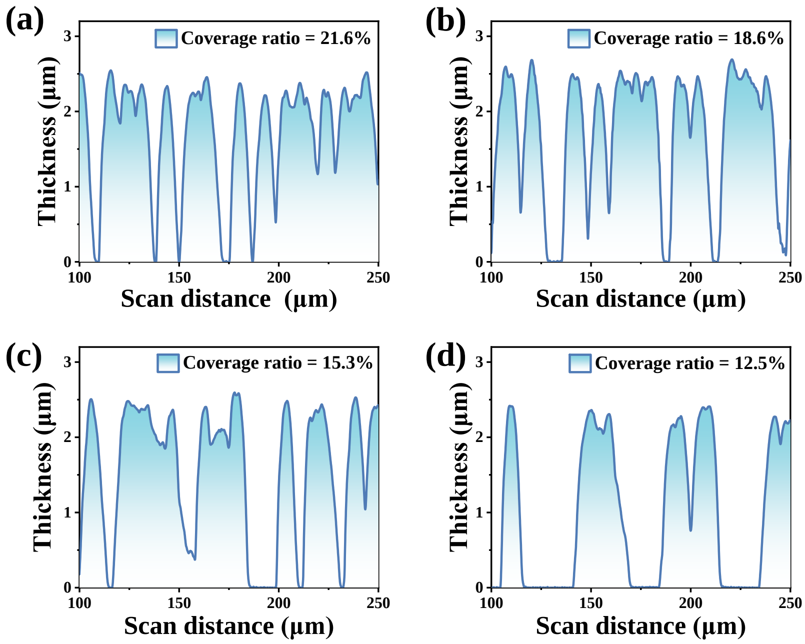

Fig. S6 Thicknesses of Cu meshes with different coverage ratios measured by step profiler

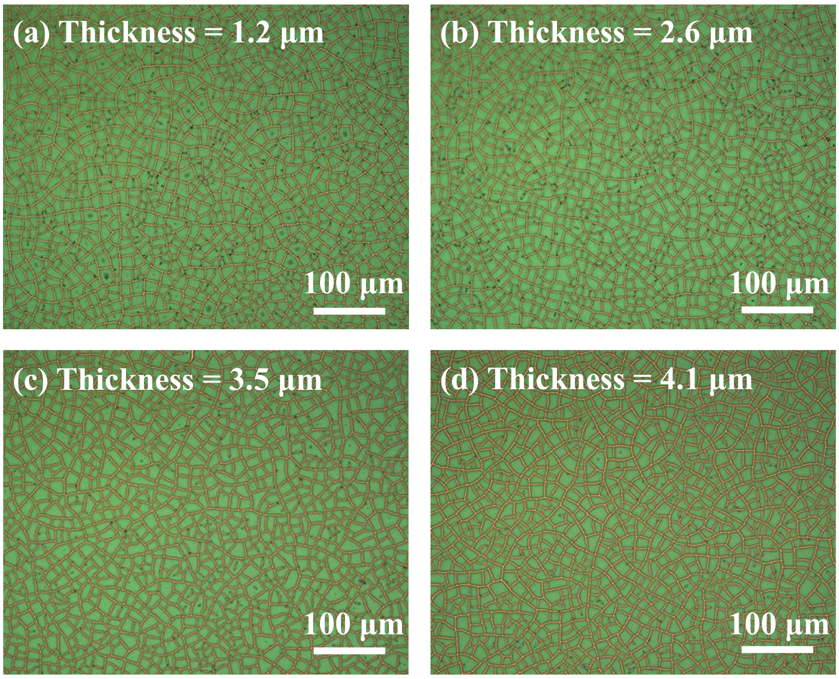

Fig. S7 OM images of Cu meshes embedded in crackle templates at different thicknesses

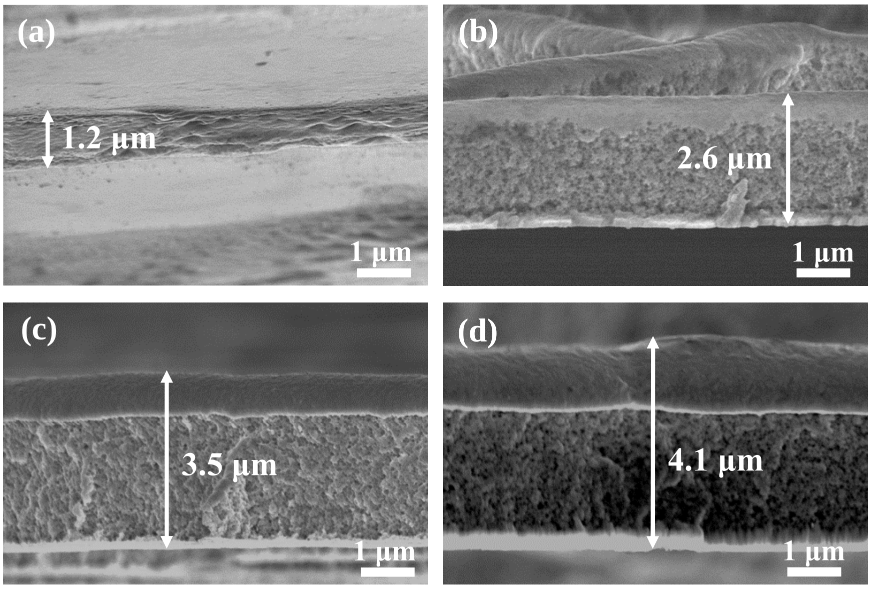

Fig. S8 Cross-sectional SEM images of Cu mesh films at different thicknesses

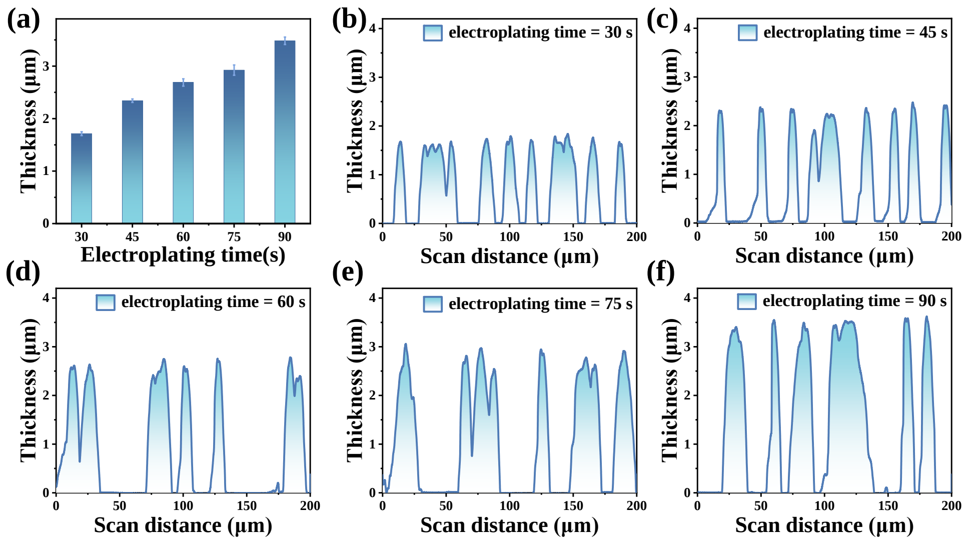

Fig. S9 a Thicknesses of the Cu mesh films at different electroplating time. **b-f** Thickness of Cu mesh films with different electroplating time measured by step profiler

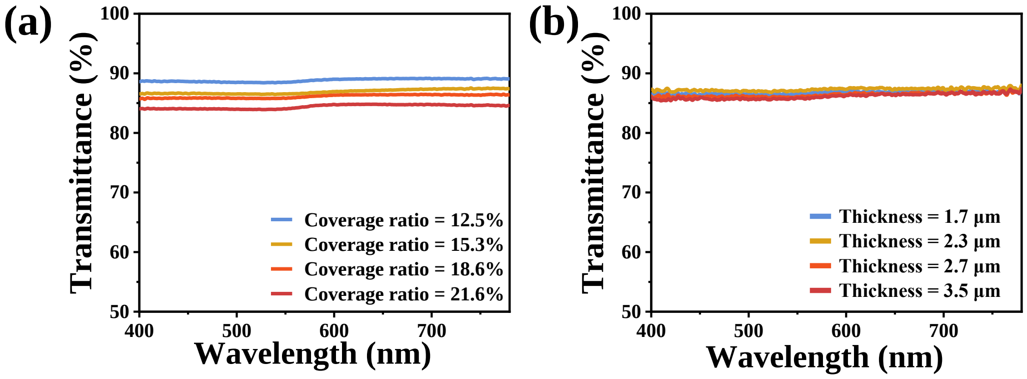

Fig. S10 a Transmittance of Cu mesh films with different coverage ratios in visible spectrum. **b** Transmittance of Cu mesh films with different thicknesses in visible spectrum

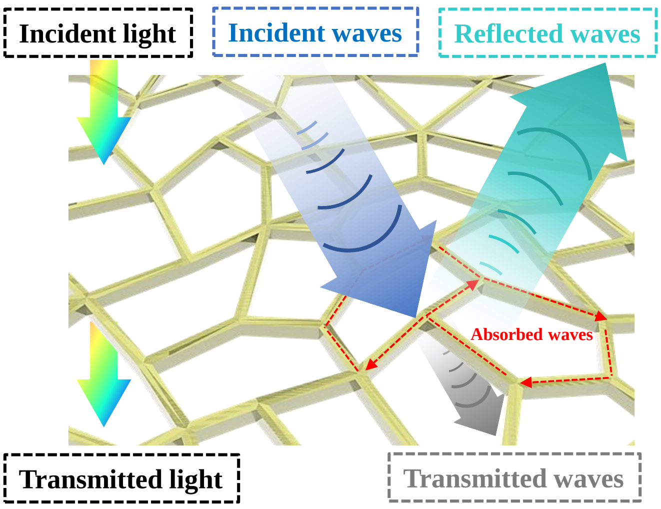

Fig. S11 Schematic diagram of the shielding mechanism for the Cu mesh

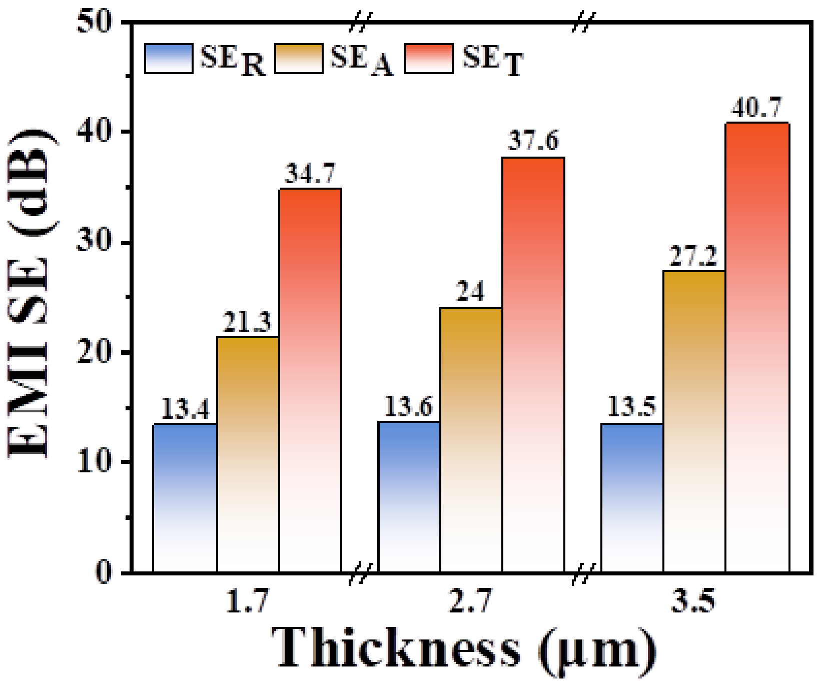

Fig. S12 Contributions of the average EM reflection and absorption to the total EMI SE for Cu mesh films with different thicknesses

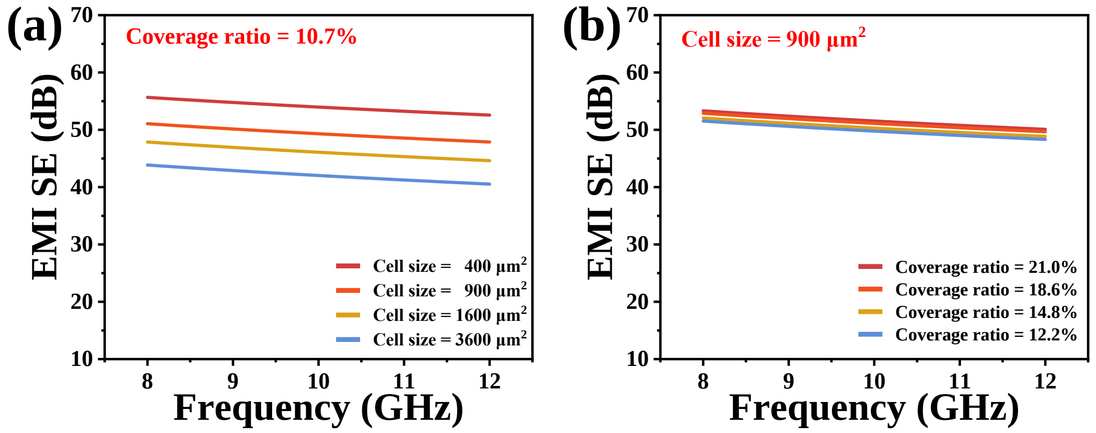

Fig. S13 a Total EMI SE of the Cu meshes (Coverage ratio = 10.7%) with different cell sizes in the X-band (8.2-12.4 GHz). **b** Total EMI SE of the Cu meshes (Cell size = 900 μm^2) with different coverage ratios in the X-band (8.2-12.4 GHz)

The EMI SE within X-band of copper mesh film was simulated by CST studio suite. The conductivity of copper was set to 5.98 × 10^7 S/m, and the permittivity of PDMS was set to 2.65. The unit area was set to 16,129 μm^2, with a total area of all cell sizes set at 14,400 μm^2, which means that all samples have a coverage ratio of around 10.7% and different cell sizes (Fig. S13a). In addition, the cell size was set to 900 μm^2, with various coverage ratio by adjusting the line width of the copper (Fig. S13b).

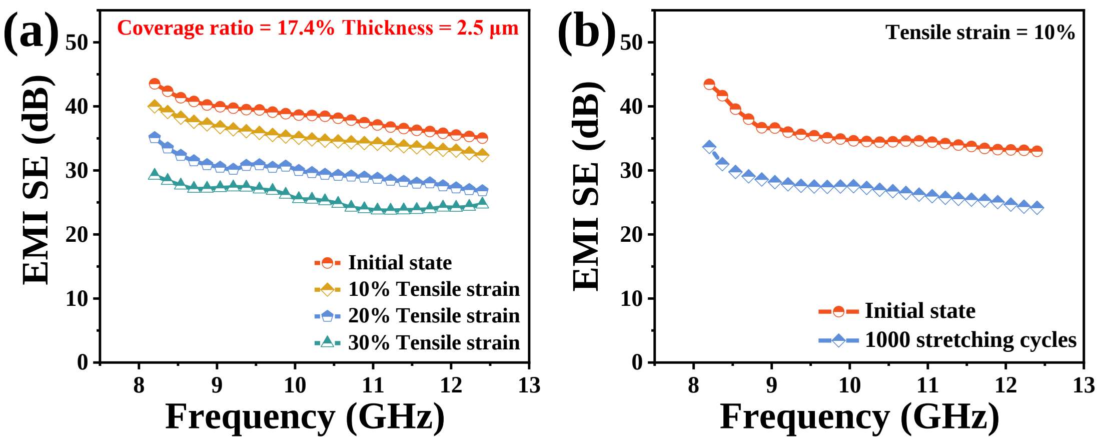

Fig. S14 EMI shielding performance of the flexible Cu mesh films (Coverage ratio = 17.4%, thickness = 2.5 μm). **a** Total EMI SE of Cu mesh at initial state and various tensile strain (10%, 20% and 30%). **b** Total EMI SE of Cu mesh at initial state and after 1000 stretching cycles at 10% tensile strain

Table S1 Parameters for Cu meshes of different coverage ratios

Parameters \ Coverage ratio	12.5%	15.3%	18.6%	21.6%
Crackle lacquer concentration (%)	100	95	90	85
Spin coating speed (rpm)	5000	5000	4500	4000
Culture-dish size (cm^2)	15 × 15	10 × 10	10 × 10	10 × 10
Temperature (°C)	35	35	35	35
Electroplating time (s)	40	50	60	75
Current density (mA cm^{-2})	20	20	20	20

Table S2 Parameters for Cu meshes of different thicknesses

Parameters \ Thickness (μm)	1.7	2.3	2.7	3.5
Crackle lacquer concentration (%)	95	95	95	95
Spin coating speed (rpm)	5000	5000	5000	5000

Culture-dish size (cm^2)	10 × 10	10 × 10	10 × 10	10 × 10
Temperature (°C)	35	35	35	35
Electroplating time (s)	30	45	60	90
Current density (mA cm^{-2})	20	20	20	20

Table S3 Comparison of sheet resistances, transmittances and method between TCFS reported in the literatures and this work

**Material**	**Method**	**Sheet resistance (Ω/□)**	**Transmittance @550nm**	**FOM**	**Refs.**
ITO	Drop-coating	20	82%	90	[1]
AgNW	Spin-coating	20.7	94.8%	337	[2]
AgNW/PSSNa	Spin-coating	10	92%	416	[3]
Graphene	Roll-to-roll and wet chemical doping	30	90%	116	[4]
Graphene/AgNW	CVD and Mayer rod coating	8	94%	750	[5]
CNT	Spin-coating	128	90%	27	[6]
MXene	Blade-coating	170	89%	18	[7]
PEDOT:PSS	Spin-coating	75	86%	32	[8]
Ag mesh	UV lithography	2.47	90.3%	1458	[9]
Cu mesh/PU	Imprinting and electroplating	0.15	82.5%	12446	[10]
Metallic mesh	Self-forming and thermal evaporation	2	76%	640	[11]

Cu mesh	Self-forming and electroplating	0.18	85.8%	13232	This work

Table S4 Comparison of the EMI SE and transmittance of this work with other materials in the literatures

**Material**	**Frequency (GHz)**	**Average EMI SE (dB)**	**Transmittance @550nm**	**Refs.**
AgNW@rGO	8.2-12.4	35	91%	[S12]
CA/AgNW/PU	8.2-12.4	21	92%	[S13]
PES/AgNW	8.2-12.4	16	85%	[S14]
AgNW	1-18	30	80%	[S15]
GNS/AgNW	12-18	28	78%	[S16]
$Ti_3C_2T_x$	8.2-12.4	4	75%	[S17]
MXene grid/AgNW	8.2-12.4	25	81%	[S18]
Ni mesh	8.2-12.4	36	93%	[S19]
Ag-Ni mesh	8.2-12.4	43	83%	[S20]
AgNW mesh	8.2-12.4	42	67%	[S21]
Cu mesh	12-18	24	82%	[S22]
Crackle template metal mesh	12-18	30	82%	[S23]
Cu mesh	8.2-12.4	41	85.8%	This work

Supplementary References

[S1] J. Y. Lee, S. T. Connor, Y. Cui, and P. Peumans, Solution-processed metal nanowire mesh transparent electrodes. Nano Lett. **8**, 689 (2008). https://doi.org/10.1021/nl073296g

[S2] J. H. Seo, I. Hwang, H. D. Um, S. Lee, K. Lee et al., Cold isostatic-pressured silver nanowire electrodes for flexible organic solar cells via room-temperature processes. Adv. Mater. **29**, 1701479 (2017). https://doi.org/10.1002/adma.201701479

[S3] Y. Sun, M. Chang, L. Meng, X. Wan, H. Gao et al., Flexible organic photovoltaics based on water-processed silver nanowire electrodes. Nat. Electron. **2**, 513 (2019). https://doi.org/10.1038/s41928-019-0315-1

[S4] S. Bae, H. Kim, Y. Lee, X. Xu, J. S. Park et al., Roll-to-roll production of 30-inch graphene films for transparent electrodes. Nat. Nanotechnol. **5**, 574 (2010). https://doi.org/10.1038/nnano.2010.132

[S5] B. Deng, P. C. Hsu, G. Chen, B. N. Chandrashekar, L. Liao et al., Roll-to-roll encapsulation of metal nanowires between graphene and plastic substrate for high-performance flexible transparent electrodes. Nano Lett. **15**, 4206 (2015). https://doi.org/10.1021/acs.nanolett.5b01531

[S6] L. Yu, C. Shearer, and J. Shapter, Recent development of carbon nanotube transparent conductive films. Chem. Rev. **116**, 13413 (2016). https://doi.org/10.1021/acs.chemrev.6b00179

[S7] T. Guo, D. Zhou, S. Deng, M. Jafarpour, J. Avaro et al., Rational design of $Ti_3C_2T_x$ MXene inks for conductive, transparent films. ACS Nano **17**, 3737 (2023). https://doi.org/10.1021/acsnano.2c11180

[S8] W. Song, X. Fan, B. Xu, F. Yan, H. Cui et al., All-solution-processed metal-oxide-free flexible organic solar cells with over 10% efficiency. Adv. Mater. **30**, 1800075 (2018). https://doi.org/10.1002/adma.201800075

[S9] M. Li, M. Zarei, K. Mohammadi, S. B. Walker, M. LeMieux et al., Silver meshes for record-performance transparent electromagnetic interference shielding. ACS Appl. Mater. Interfaces **15**, 30591 (2023). https://doi.org/10.1021/acsami.3c02088

[S10] X. Chen, Y. Yin, W. Yuan, S. Nie, Y. Lin et al., Transparent thermotherapeutic skin patch based on highly conductive and stretchable copper mesh heater. Adv. Electron. Mater. **7**, 2100611 (2021). https://doi.org/10.1002/aelm.202100611

[S11] B. Han, K. Pei, Y. Huang, X. Zhang, Q. Rong et al., Uniform self-forming metallic network as a high-performance transparent conductive electrode. Adv. Mater. **26**, 873 (2014). https://doi.org/10.1002/adma.201302950

[S12] Y. Yang, S. Chen, W. Li, P. Li, J. Ma et al., Reduced graphene oxide conformally wrapped silver nanowire networks for flexible transparent heating and electromagnetic interference shielding. ACS Nano **14**, 8754 (2020). https://doi.org/10.1021/acsnano.0c03337

[S13] L. C. Jia, D. X. Yan, X. Liu, R. Ma, H. Y. Wu et al., Highly efficient and reliable transparent electromagnetic interference shielding film. ACS Appl. Mater. Interfaces **10**, 11941 (2018) https://doi.org/10.1021/acsami.8b00492

[S14] M. Hu, J. Gao, Y. Dong, K. Li, G. Shan et al., Flexible transparent pes/silver nanowires/pet sandwich-structured film for high-efficiency electromagnetic interference shielding. Langmuir **28**, 7101 (2012). https://doi.org/10.1021/la300720y

[S15] X. Zhang, Y. Zhong, and Y. Yan, Electrical, mechanical, and electromagnetic shielding properties of silver nanowire-based transparent conductive films. Phys. Status Solidi Appl. Mater. Sci. **215**, 1800014 (2018). https://doi.org/10.1002/pssa.201800014

[S16] N. Zhang, Z. Wang, R. Song, Q. Wang, H. Chen et al., Flexible and transparent graphene/silver-nanowires composite film for high electromagnetic interference shielding effectiveness. Sci. Bull. **64**, 540 (2019). https://doi.org/10.1016/j.scib.2019.03.028

[S17] T. Yun, H. Kim, A. Iqbal, Y. S. Cho, G. S. Lee et al., Electromagnetic shielding of monolayer MXene assemblies. Adv. Mater. **32**, 1906769 (2020). https://doi.org/10.1002/adma.201906769

[S18] M. Jin, W. Chen, L. X. Liu, H. Bin Zhang, L. Ye et al., Transparent, conductive and flexible MXene grid/silver nanowire hierarchical films for high-performance electromagnetic interference shielding. J. Mater. Chem. A **10**, 14364 (2022). https://doi.org/10.1039/d2ta03689d

[S19] Z. Jiang, W. Huang, L. Chen, and Y. Liu, Ultrathin, lightweight, and freestanding metallic mesh for transparent electromagnetic interference shielding. Opt. Express **27**, 24194 (2019). https://doi.org/10.1364/oe.27.024194

[S20] S. Shen, S.-Y. Chen, D.-Y. Zhang, and Y.-H. Liu, High-performance composite Ag-Ni mesh based flexible transparent conductive film as multifunctional devices. Opt. Express **26**, 27545 (2018). https://doi.org/10.1364/oe.26.027545

[S21] J. Gu, S. Hu, H. Ji, H. Feng, W. Zhao et al., Multi-layer silver nanowire/polyethylene terephthalate mesh structure for highly efficient transparent electromagnetic interference shielding. Nanotechnology **31**, 185303 (2020). https://doi.org/10.1088/1361-6528/ab6d9d

[S22] Y. Han, H. Zhong, N. Liu, Y. Liu, J. Lin et al., In situ surface oxidized copper mesh electrodes for high-performance transparent electrical heating and electromagnetic interference shielding. Adv. Electron. Mater. **4**, 1800156 (2018) https://doi.org/10.1002/aelm.201800156

[S23] Y. Han, J. Lin, Y. Liu, H. Fu, Y. Ma et al., Crackle template based metallic mesh with highly homogeneous light transmission for high-performance transparent EMI shielding. Sci. Rep. **6**, 25601 (2016) https://doi.org/10.1038/srep25601
